# Hematopoietic and stromal DMP1-Cre labeled cells form a unique niche in the bone marrow

**DOI:** 10.1038/s41598-023-49713-x

**Published:** 2023-12-16

**Authors:** Sierra H. Root, Brya G. Matthews, Elena Torreggiani, Hector Leonardo Aguila, Ivo Kalajzic

**Affiliations:** 1 Center for Regenerative Medicine and Skeletal Development, MC 3705, School of Dental Medicine, UConn Health, 263 Farmington Ave, Farmington, CT 06030 USA; 2 Division of Pediatric Dentistry, MC1610, School of Dental Medicine, UConn Health, 263 Farmington Ave, Farmington, CT 06030 USA; 3 Department of Molecular Medicine and Pathology, University of Auckland, Auckland, New Zealand; 4 Department of Immunology, UConn Health, Farmington, USA

**Keywords:** Cell biology, Developmental biology

## Abstract

Skeletogenesis and hematopoiesis are interdependent. Niches form between cells of both lineages where microenvironmental cues support specific lineage commitment. Because of the complex topography of bone marrow (BM), the identity and function of cells within specialized niches has not been fully elucidated. Dentin Matrix Protein 1 (DMP1)-Cre mice have been utilized in bone studies as mature osteoblasts and osteocytes express DMP1. DMP1 has been identified in CXCL12^+^ cells and an undefined CD45^+^ population. We crossed DMP1-Cre with Ai9 reporter mice and analyzed the tdTomato^+^ (tdT^+^) population in BM and secondary hematopoietic organs. CD45^+^tdT^+^ express myeloid markers including CD11b and are established early in ontogeny. CD45^+^tdT^+^ cells phagocytose, respond to LPS and are radioresistant. Depletion of macrophages caused a significant decrease in tdT^+^CD11b^+^ myeloid populations. A subset of CD45^+^tdT^+^ cells may be erythroid island macrophages (EIM) which are depleted after G-CSF treatment. tdT^+^CXCL12^+^ cells are in direct contact with F4/80 macrophages, express RANKL and form a niche with B220^+^ B cells. A population of resident cells within the thymus are tdT^+^ and express myeloid markers and RANKL. In conclusion, in addition to targeting osteoblast/osteocytes, DMP1-Cre labels unique cell populations of macrophage and stromal cells within BM and thymus niches and expresses key microenvironmental factors.

## Introduction

The cells involved in skeletogenesis and medullary hematopoiesis arise from two distinct lineages whose interaction begins at the onset of vascularization and establishment of the bone marrow (BM) niche within fetal long bones^1^. Although extramedullary hematopoietic organs such as the spleen, liver and peripheral blood maintain small numbers of progenitors throughout adult life, these organs are more active during disease states and during early development. BM contains cells of the hematopoietic lineage as well nerves, stromal cells, vascular endothelial cells and perivascular cells which are encased by osteoblasts and osteocytes embedded in mineralized matrix. The complexity of all these cell types within specialized niches allows for proper control of hematopoietic maintenance and differentiation as well as bone formation^2–8^. Within the complex BM topography exists distinct resident macrophages each with their own supporting roles and niches. Specific resident macrophage populations include osteal macrophages (osteomacs), hematopoietic stem cell (HSC) niche macrophages and erythroid island macrophages (EIM)^4,9–11^. Another important niche supporting cell type are CXCL12 abundant reticular (CAR) cells which are located within both vascular and endosteal niches where they maintain quiescent HSCs via a CXCL12–CXCR4 axis^12^. CAR cells have been shown to be important regulators of B cell lymphopoiesis by expression of the key lymphoid cytokine interleukin 7 (IL-7)^13^. More recently adipogenic CAR cells have been shown to provide hematopoietic supporting cytokines^14^. Furthermore, Adipoq-Cre-labeled CAR cells have been shown to express myeloid/macrophage supporting cytokines including M-CSF and RANKL which directly support development of resident macrophages and osteoclastogenesis^15,16^.

Dentin matrix protein 1 (DMP1) was initially identified as an acidic extracellular matrix protein and a member of the small integrin-binding ligand N-linked glycoprotein (SIBLING) family in odontoblasts^17,18^. DMP1 has been further identified as a marker gene for mature osteoblasts and osteocytes^19–21^ and is critical for proper phosphate metabolism and mineralization during bone development^22,23^. Furthermore, a chondroitin sulfate enriched proteoglycan results after glycosylation of the N-terminal fragment of DMP1 which further regulates osteogenesis^24,25^. DMP1 has also been shown to reside in the nucleus where it regulates gene expression of osteoblast genes before it is exported to the extracellular matrix during osteoblast maturation^26,27^. Importantly extracellular DMP1 has also be shown to interact with alphaVbeta3 integrins in mesenchymal cells which activates MAPK pathways^28^.

DMP1 has been identified in tissues outside of the odontoblast/osteoblast lineages including astrocytes in the brain, gut mesenchyme, skeletal muscle and kidney, suggesting that DMP1 has a role in nonmineralized tissues^29–31^. We and others have identified a population of DMP1-Cre labeled cells in BM when crossed with Ai9, but little attention has been placed on the identity and function of these cells including if they provide support to the microenvironment^30,32–34^. Initial evaluations demonstrated these cells reside near blood vessels and a portion of them are positive for the chemokine CXCL12 suggesting they are CAR cells. Previously an identified population of DMP1-CreERT2/Ai9 and DMP1-GFP^+^ cells were found to be hematopoietic based on CD45 expression^33,35^.

DMP1-Cre has been utilized to genetically target osteoblasts and osteocytes and described osteocytes as the major producers of the key osteoclast differentiation factor RANKL^36,37^ and as a key regulator of B cell development^38^ without considering the function of DMP1-Cre cells in BM. Ablation of osteocytes using DMP1 promoter-driven diphtheria toxin receptor (DTR) demonstrated severe lymphopenia and thymic atrophy which was attributed to osteocyte depletion^39^. Therefore, in order to understand if DMP1-Cre labeled cells in bone marrow have a role in hematopoiesis and/or skeletogenesis, we crossed DMP1-Cre with Ai9 reporter mice and evaluated labeled cells in bone marrow and secondary hematopoietic organs. We hypothesized these cells have a role in supporting the bone marrow microenvironment and should be considered when using DMP1-Cre to target osteocytes/osteoblasts.

## Results

### Identification of hematopoietic and stromal DMP1-Cre/Ai9 labeled cells in bone marrow

**Figure 1:**
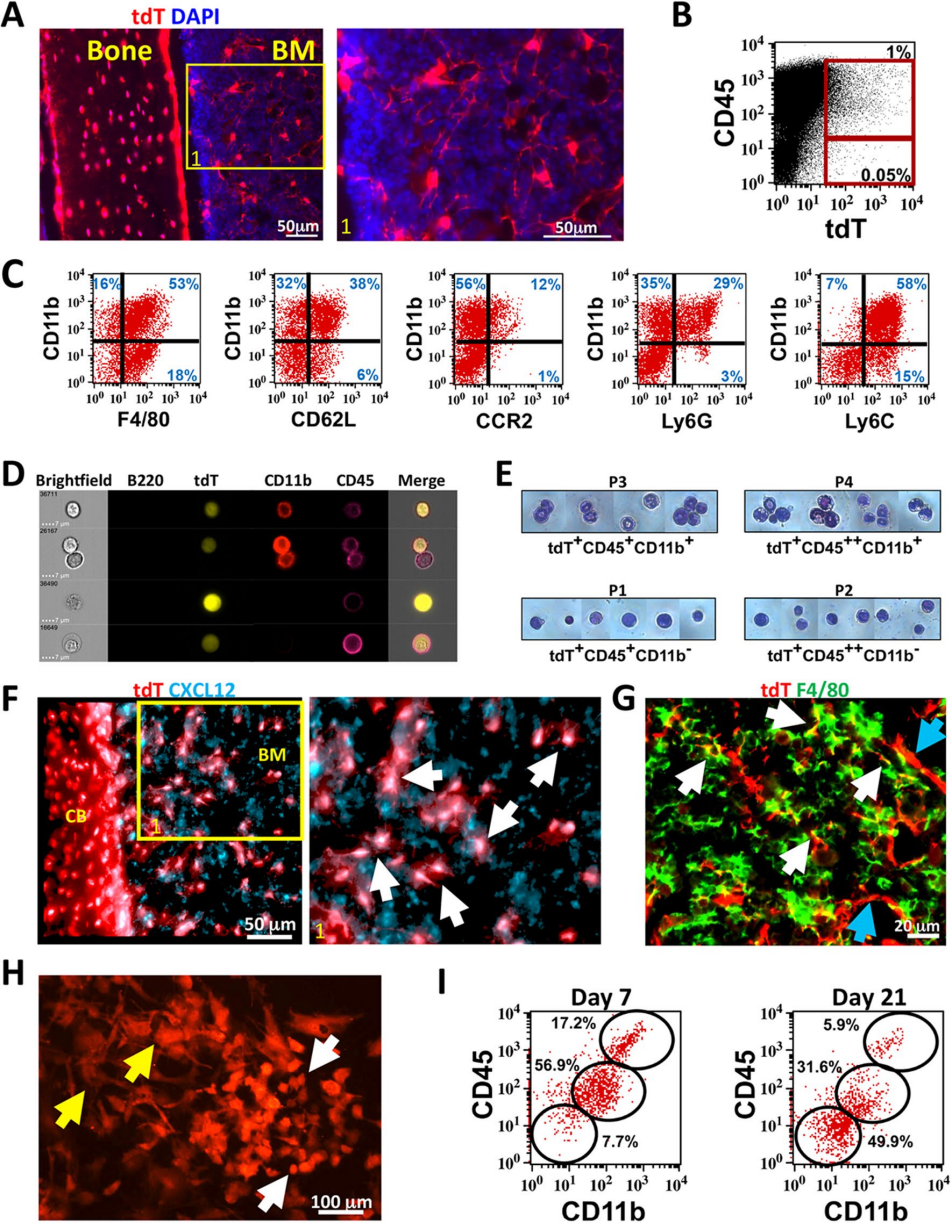
DMP1-Cre/Ai9 labeled hematopoietic and stromal populations in bone marrow. (**A**) Femoral frozen histology of 8 week old DMP1-Cre/Ai9 cortical bone and bone marrow (BM). Red-tdT, Blue-DAPI. Yellow box indicates magnified area of BM. (**B**) Flow cytometric dot plot of bone marrow flush stained for CD45 and tdT. (**C**) Flow cytometry analysis of CD45^+^tdT^+^ population for myeloid markers F4/80, CD62L, CCR2, Ly6G and Ly6C with CD11b. (**D**) Image stream analysis of tdT^+^ (yellow) bone marrow cells stained for B220 (green), CD11b (red) and CD45 (magenta). Gating strategy show in Supplemental Fig. 2 to identify single tdT^+^CD45^+^ cells. (**E**) Bone marrow tdT^+^ cells from 12 week old mice were FACS sorted based on 4 populations identified (P1–P4) for CD45 and CD11b expression and cytospun onto glass slides (gating strategy show in Supplemental Fig. 1). Giemsa staining of each population is shown. (**F**) 3D rendering of Z stacks from DMP1-Cre/Ai9 cortical bone (CB) and bone marrow (BM) stained for CXCL12 (cyan). Enlarged area shown, white arrows point to colocalization (white) of tdT and CXCL12. (**G**) Femoral frozen histology of DMP1-Cre/Ai9 bone marrow stained for F4/80 (green). White arrows—F4/80^+^tdT^+^ macrophages; Blue arrows—F4/80^−^tdT^+^ stromal cells. (**H**,**I**) Bone marrow flushes were plated with open B6 femurs to allow for supporting factors in the media. (**H**) tdT cells were imaged at day 7 of culture. Yellow arrow—stromal cells, White arrow—hematopoietic cells. (**I**) Flow cytometry analysis of gated tdT^+^ cells at day 7 and day 21 of culture for CD45 and CD11b expression.

To study non-osteogenic DMP1-Cre labeled cells in BM, we crossed 10 kb DMP1-Cre with Ai9 reporter animals (DMP1-Cre/Ai9). Osteocytes in cortical bone, endosteal osteoblasts and cells in BM are labeled with tdTomato (tdT) (Fig. 1A and Supplemental Fig. 1A). Flow cytometry showed that less than 1% of CD45^+^ cells were tdT^+^ (Fig. 1B and Supplemental Fig. 1B). A rare CD45^−^TdT^+^ stromal population is also evident. Initial evaluation of the CD45^+^tdT^+^ by flow cytometry using antibodies to identify CD3^+^ T cells, B220^+^ B cells, and CD11b^+^ myeloid cells demonstrated that most of these cells are positive for the myeloid marker CD11b (Supplemental Fig. 1C). When CD45^+^tdT^+^ cells were further analyzed by flow cytometry using a combination of myeloid specific markers, a heterogeneous population of myeloid cells was observed expressing various degrees of macrophage/monocyte markers F4/80, CD62L, CCR2, Ly6G, and Ly6C with CD11b (Fig. 1C). Recent studies have demonstrated that mature tissue-resident macrophages do not survive most cell processing procedures used for flow cytometry, but their fragments can attach to other cells, confounding results^40^. We evaluated the possibility of fragmentation using Image Stream analysis. We confirmed the presence of a rare CD45^+^tdT^+^ population consisting of CD11b^+^ and CD11b^−^ cells by Image Stream analysis (Fig. 1D and Supplemental Fig. 2A). Occasional tdT^+^ fragments were seen with CD45^+^CD11b^+^ cells or with B220^+^ cells but this did not represent the majority of tdT^+^ cells with Image Stream analysis (Supplemental Fig. 2B). A triple cell sorting strategy to obtain four pure populations of tdT^+^ cells based on CD45 and CD11b expression was done (Supplemental Fig. 1D–F). Morphological heterogeneity of sorted cell populations was observed (Fig. 1E). CD45^+^CD11b^−^ appeared to have more of a monocyte/myelocyte appearance based on size and nuclear shape^41^. tdT^+^ cells that were CD45^+^CD11b^+^ consisted of cells with banded and mature neutrophil appearance including cells with phagocytic vacuoles.

Previously a subset of DMP1-Cre/Ai9 cells evaluated by flow cytometry were shown to express CXCL12^32^. We evaluated femoral sections stained with CXCL12 antibody and confirmed tdT^+^ stromal population likely represents CAR cells (Fig. 1F and Supplemental Fig. 3). By histology of DMP1/Cre/Ai9 femurs, we confirmed the presence of tdT^+^F4/80^+^ macrophages (Fig. 1G, white arrows) as well as tdT^+^F4/80^−^ stromal cells in the BM (Fig. 1G, blue arrows). BM cells grown in vitro demonstrate two populations of tdT^+^ cells (Fig. 1H)—tdT^+^ cells consistent with hematopoietic morphology (white arrows) and fibroblastic stromal like cells (yellow arrow). Based on flow cytometry, the majority of tdT^+^ cells at day 7 of culture were CD45^+^CD11b^+^, but this decreased over time with a non-hematopoietic population becoming the major cell type in culture by day 21 (Fig. 1I). Collectively, our data shows that DMP1-Cre/Ai9 labeled BM contains both a CD45^+^tdT^+^ myeloid population, and a CD45^−^tdT^+^ stromal population that express CXCL12.

### Hematopoietic DMP1-Cre/Ai9 labeled cells during development and in secondary hematopoietic organs

**Figure 2:**
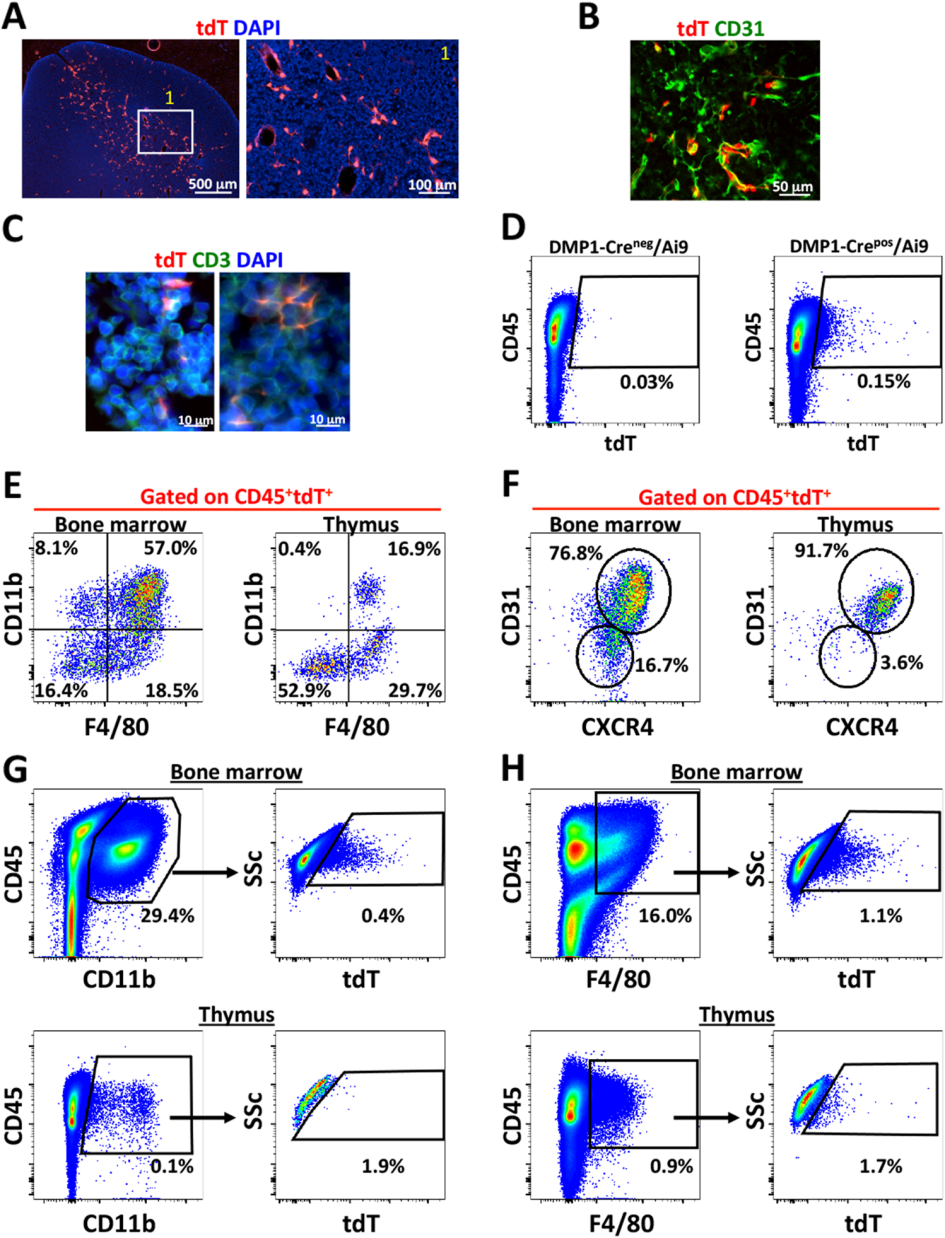
DMP1-Cre/Ai9 labeled cells in thymus. (**A**,**B**) Frozen histology of 8 week old DMP1-Cre/Ai9 thymus for tdT (red), DAPI (blue) and (**B**) CD31 staining (green). White box indicates magnified area. (**C**) 10 day old thymus stained for CD3 (green) (**D**) Flow cytometric of single cell suspensions of thymus from 4 month old Cre− and Cre+ animals. Cell gate shown for CD45^+^tdT^+^ cells. (**E**) FACs plots of CD45^+^tdT^+^ bone marrow and thymus cells for CD11b and F4/80 and (**F**) CD31 and CXCR4 expression. (**G**) Flow cytometric analysis of tdT^+^ cells in bone marrow and thymus gated from CD45^+^CD11b^+^ cells. (**H**) Flow cytometric analysis of tdT^+^ cells in bone marrow and thymus gated from CD45^+^F4/80^+^ cells.

We evaluated DMP1-Cre/Ai9 labeled cells in BM during development and found that CD45^+^tdT^+^ cells are present around E15.5. Most CD45^+^tdT^+^ cells are CD11b^+^ at E15.5, but this decreases with age (Supplemental Fig. 4A–D). We did not find tdT^+^ cells in secondary hematopoietic organs during development (Supplemental Fig. 5A–C). Adult spleen and blood had a population of tdT^+^ cells representing 0.01% of total population however liver never showed tdT^+^ cells by flow cytometry. However, adult thymus demonstrated a population of CD45^+^tdT^+^ cells (Supplemental Fig. 5C). By histology, tdT^+^ cells were seen primarily in the medulla, around blood vessels (Fig. 2A,B) and close to CD3^+^ T cells in the cortex (Fig. 2C). Flow cytometric analysis of total thymic cells had an average 0.15% CD45^+^tdT^+^ cells (Fig. 2D). CD45^+^tdT^+^ cells in BM and thymus both contained CD11b^+^F4/80^+^ macrophage populations, but these cells are much more abundant in BM (Fig. 2E). Resident thymic macrophages include both CD11b^+^ F4/80^+^ and CD11b^−^F4/80^+^ populations^42^. The CD45^+^tdT^+^ thymus population contains 29.7% CD11b^−^F4/80^+^ macrophages (Fig. 2E). CD45^+^tdT^+^ cells in thymus were 91.7% CD31^+^CXCR4^+^ compared to 76.8% in bone marrow (Fig. 2F). tdT^+^ cells represented a rare subset of both CD11b^+^ and F4/80^+^ myeloid cells in the BM and thymus (Fig. 2G,H). These results suggest that BM and thymic tdT^+^ cells most likely represent a specific resident macrophage within their respective organs.

Because of the primarily medullary location of CD45^+^tdT^+^ cells in thymus, we wanted to exclude the possibility tdT^+^ labeled cells being medullary thymic epithelial cells (mTECS). By using specific digestion protocols, mTECs can be isolated and should not be CD45^+^ or express myeloid markers but instead express the autoimmune regulator (AIRE) protein^43^. The role of AIRE^+^ mTECs cells in thymus is to maintain immune tolerance by clearing auto-reactive T cells^44–48^. Therefore, thymi were processed using digestion methods to isolate fractions containing mTECs and compared to BM by flow cytometry (Supplemental Fig. 6A,B). Using this method, we enriched the CD45^+^tdT^+^ fraction (from 0.1 to 0.29% when compared to total thymus preps), but flow cytometry indicated that tdT^+^ cells are not mTECS, based on CD45 expression or other myeloid markers including CD11b, CD31 and CD44. We further verified that tdT^+^ cells in thymus were not mTECs by AIRE staining. BM and thymic CD45^+^tdT^+^ cells showed no AIRE expression by flow cytometry and thymus showed no double positive cells by histology (Supplemental Fig. 6C,D).

### CD11b^+^DMP1-Cre/Ai9 labeled cells include macrophages

**Figure 3:**
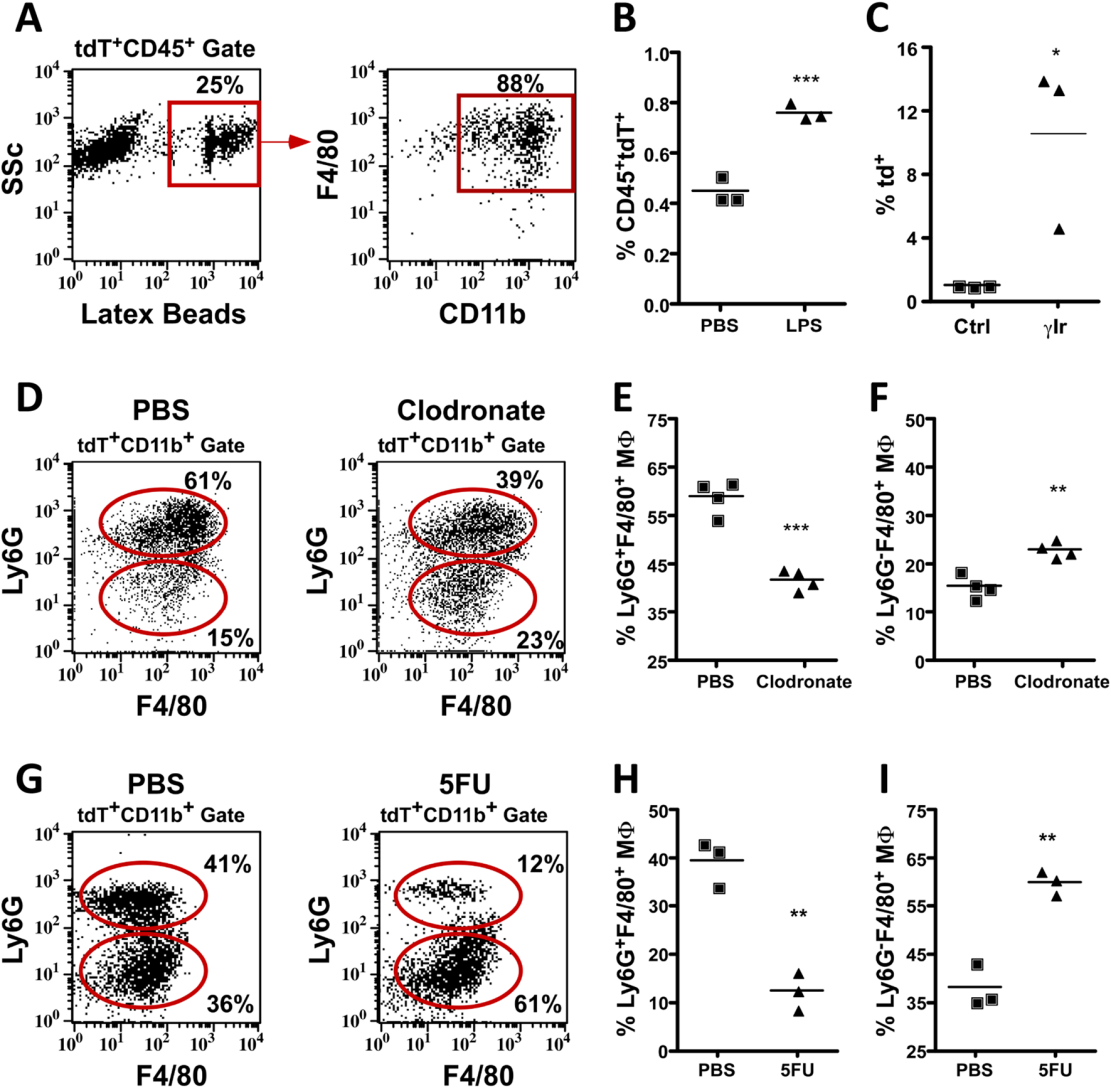
Macrophage properties of DMP1-Cre/Ai9 labeled hematopoietic fraction in bone marrow. (**A**) Bone marrow flush incubated with 1 mM latex YG beads and then stained for CD11b and F4/80. FACs plots are first gated on CD45^+^tdT^+^ population, then fluorescent latex beads. (**B**) Flow analysis of 12 week old mice quantified for the CD45^+^tdT^+^ population in mice treated for 3 h with 25 mg LPS, ***p = 0.0009. (**C**) Percent tdT^+^ cells in 3 month old control (Ctrl) and 6 days post lethal gamma irradiation (γIr) dose (950 rads) *p = 0.0327 (**D**–**F**) 8 week old DMP1-Cre/Ai9 animals 36 h after macrophage depletion with clodronate loaded liposomes compared to control. Representative flow cytometry analysis of the macrophage (MΦ) tdT^+^CD11b^+^ population for F4/80 and Ly6G which is quantified in (**E**) for percent Ly6G^−^F4/80^+^ MΦ cells, **p = 0.002 and (**F**) percent Ly6G^+^F4/80^+^ MΦ cells, ***p = 0.0001. (**G**–**I**) 8 week old DMP1-Cre/Ai9 animals 4 days after single dose of 5-FU. Representative flow cytometry analysis of macrophages within the tdT^+^CD11b^+^ population for F4/80 and Ly6G which is quantified in (**H**) for percent Ly6G^−^F4/80^+^ MΦ cells, **p = 0.0018, and (**I**) percent Ly6G^+^F4/80^+^ MΦ cells, **p = 0.0016. n = 3–4 and SEM is shown in all graphs.

In order to further define the identity and function of the myeloid DMP1-Cre/Ai9 labeled population, cells were analyzed for their phagocytic ability and their response to environmental changes. Ex vivo, 25% of CD45^+^tdT^+^ cells phagocytosed fluorescently labeled beads (Fig. 3A). Most of the tdT^+^ cells capable of phagocytosis were CD11b^+^F4/80^+^. Next, we evaluated the response of tdT^+^ cells to bacterial lipopolysaccharide (LPS). DMP1-Cre/Ai9 animals showed a significant increase in CD45^+^tdT^+^ cells 3 h after LPS administration (Fig. 3B). BM macrophages are resistant to lethal doses of irradiation^49–51^, so we administered lethal irradiation to DMP1-Cre/Ai9 animals. Six days post irradiation bone marrow cellularity decreased by 93.8% and the percent of tdT^+^ cells was significantly higher when compared to controls (Supplemental Fig. 7A and Fig. 3C).

We used two different methods to deplete macrophages and evaluate the effects on the CD45^+^tdT^+^ population. Clodronate-loaded liposomes efficiently deplete macrophages in vivo^10,52,53^. We assessed the effect of clodronate-loaded liposome treatment on cells expressing CD11b, Ly6G, and F4/80 in DMP1-Cre/Ai9 mice by flow cytometry (Fig. 3D–F). Bone marrow cellularity decreased by 36.7% and tdT^+^CD11b^+^ cells showed a significant reduction in Ly6G^+^F4/80^+^ and increase in Ly6G^−^F4/80^+^ cells (Supplemental Fig. 7B and Fig. 3D–F). Myeloablative agents such as 5-fluorouracil (5FU) have shown to collapse the BM niche including resident macrophages which in turn causes egress of HSC to the periphery^54^. BM was analyzed by flow cytometry 4 days after 5FU treatment (Fig. 3G–I). Bone marrow cellularity decreased by 96.2% and a significant reduction in Ly6G^+^F4/80^+^ and increase in Ly6G^−^F4/80^+^ cells occurred within tdT^+^CD11b^+^ cells (Supplemental Fig. 7C and Fig. 3G–I). Due to potential fragmentation of macrophages our data could also include a population of neutrophils bound to tdT^+^ macrophage remnants that is depleted upon these treatments.

### A subset of myeloid DMP1-Cre/Ai9 labeled cells are erythroid island macrophages

Although BM-resident macrophages share similar cell surface markers, certain populations can be identified with specific marker combinations. In order to determine which Dmp1-Cre/Ai9 macrophage population was being identified, flow cytometry was done using various marker combinations in order to identify osteomacs^10^, HSC niche macrophages^54^ and erythroid island macrophages (EIM) which can be enriched from isolated BM and express cell surface markers including CD11b, F4/80, Ly6G, CD169, ER-HR3, VCAM1 and CD51^52,55,56^. One of the main functions of this central macrophage is to support maturation of erythroblasts. EIM support developing reticulocytes during enucleation by efferocytosis of extruded nuclei^11,57^. Due to the myeloid marker expression pattern of CD45^+^tdT^+^ cells (Fig. 1C) we aimed to determine if these cells represented EIMs.

**Figure 4:**
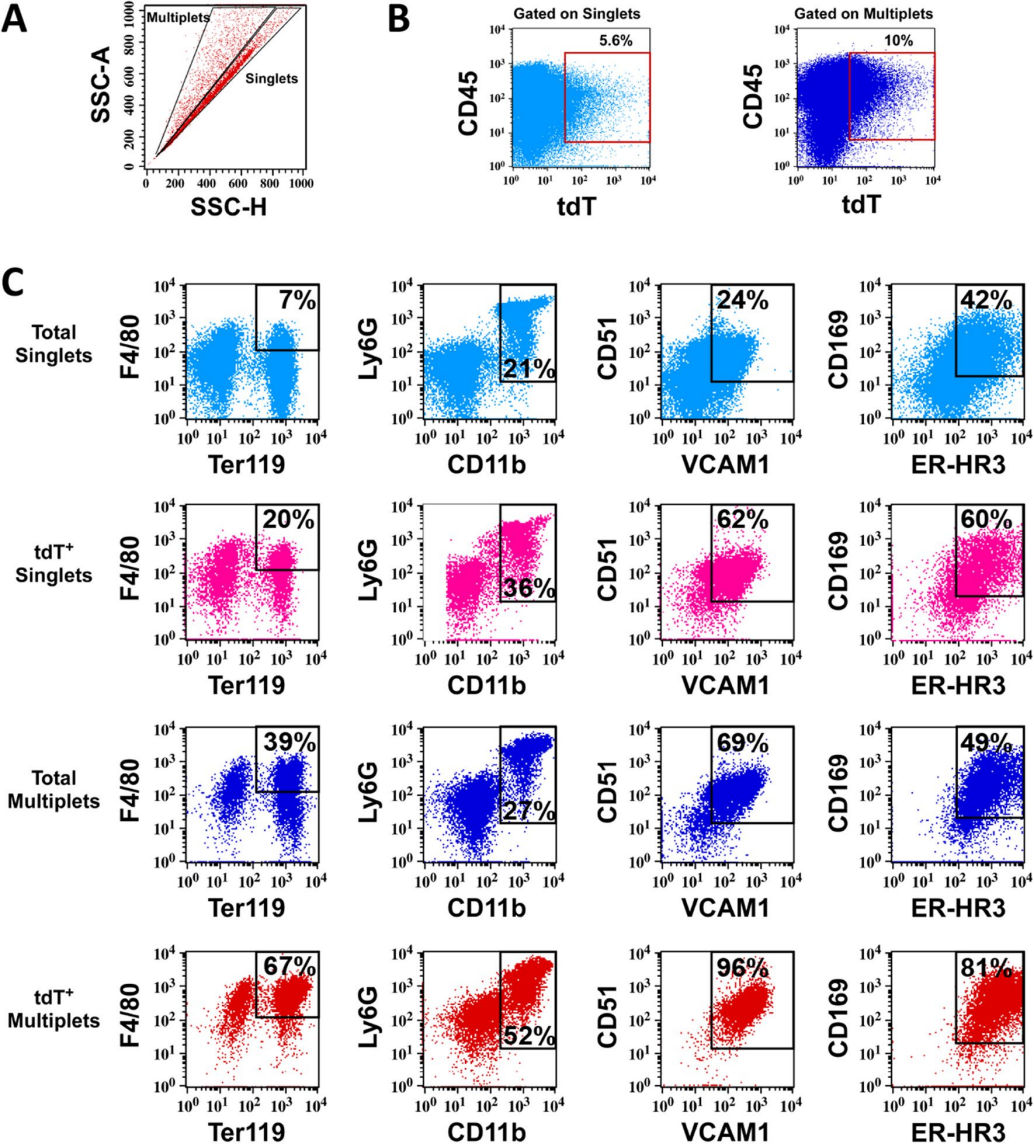
DMP1-Cre/Ai9 labeled myeloid cells are enriched in erythroid islands and express erythroid island macrophage (EIM) markers. (**A**–**C**) BM from 8 week old DMP1-Cre/Ai9 animals were processed to obtain and analyze intact blood islands by flow cytometry, n = 3 (**A**) SSC-A versus SSC-H identifies Singlets and Multiplets which were gated and analyzed in the context of (**B**) CD45 and tdT. (**C**) Blood islands were further identified by flow cytometry as F4/80^+^Ter119^+^Multiplet events and EIM by additional markers Ly6G, CD11b, CD51, VCAM1, CD169, and ER-HR3. As a control, total Singlets and Multiplets are shown for these markers. Enrichment of blood islands and EIM markers occurs within CD45^+^tdT^+^Multiplets gates.

We first validated enrichment of the myeloid CD45^+^tdT^+^ population using two separate methods to isolate EIM (Supplemental Fig. 8A)^58,59^. Since by histology tdT^+^ cells are in proximity to Ter119^+^ cells (Supplemental Fig. 8B), we utilized a previously described method to analyze intact erythroid blood islands (EBI) by flow cytometry (Fig. 4)^55,60^. Clusters of cells (multiplets) can be found by analyzing side scatter properties outside of the singlet gate, and EBIs containing EIMs by co-staining of Ter119 and F4/80. We confirmed enrichment of CD45^+^tdT^+^ events in the multiplet population (Fig. 4A,B). We next analyzed CD45^+^tdT^+^Multiplets for F4/80 and Ter119 to confirm the presence of tdT^+^ blood islands. CD45^+^tdT^+^Multiplets showed enrichment of EIM markers Ly6G, CD51, VCAM1, CD169 and ER-HR3 (Fig. 4C).

**Figure 5:**
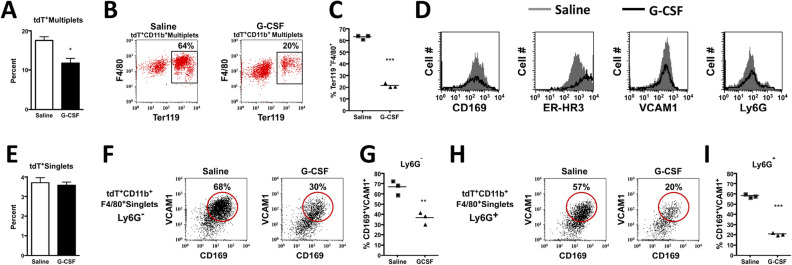
Effect of G-CSF on the DMP1-Cre/Ai9 labeled erythroid blood islands and EIMs. G-SCF was administered to 5 week old DMP1-Cre/Ai9 animals at 50 mg/kg, twice a day for 3 days. BM was processed for intact blood islands, n = 3. (**A**) Quantified flow data of percent tdT^+^Multiplets in PBS and G-CSF treated mice, *p = 0.0218. (**B**) Representative FACs plots of tdT^+^CD11b^+^Multiplets with cells double positive for F4/80 and Ter119 which is quantified in (**C**) ***p = < 0.0001. (**D**) Histograms of G-CSF (black line) and PBS (grey filled line) treated mice for expression of EIM markers within the tdT^+^CD11b^+^Ter119^+^F4/80^+^Multiplet gates. (**E**–**I**) BM flushes with niche disruption and RBC lysis after G-CSF treatment. (**E**) Quantified flow data of percent tdT^+^Singlets in PBS and G-CSF treated mice. (**F**) Representative FACs plots of tdT^+^CD11b^+^F4/80^+^Ly6G^−^Singlets with cells double positive for CD169 and VCAM1 which is quantified in (**G**) **p = 0.0043. (**H**) Representative FACs plots of tdT^+^CD11b^+^F4/80^+^Ly6^+^Singlets which is quantified in (**I**) ***p < 0.0001. SEM is shown in all graphs.

Granulocyte colony stimulating factor (G-CSF) mobilizes HSCs to the periphery by disturbing niches within the bone marrow^61^ including those of resident macrophages^10,52,62^. G-CSF causes loss of EIM resulting in a profound decrease in medullary erythropoiesis^52^. We therefore treated mice for 3 days with G-CSF and analyzed blood islands and EIM by flow cytometry. tdT^+^Multiplets and tdT^+^CD11b^+^Multiplets double positive for F4/80 and Ter119 (blood islands) significantly decreased after G-CSF treatment (Fig. 5A–C). tdT^+^ EIM cell numbers within blood islands expressing CD169, ER-HR3, VCAM1 and Ly6G all decreased after G-CSF treatment (Fig. 5D). There was no significant difference in the proportion of tdT^+^Singlets after G-CSF treatment (Fig. 5E). However, both tdT^+^CD11b^+^F4/80^+^Ly6G^−^ and Ly6G^+^ resident macrophage subsets showed significant decreases in VCAM1^+^CD169^+^ double positive cells after G-CSF treatment (Fig. 5F–I) demonstrating the loss of multiple resident macrophages after niche disruption.

### Endogenous DMP1 expression and lineage tracing of DMP1

**Figure 6:**
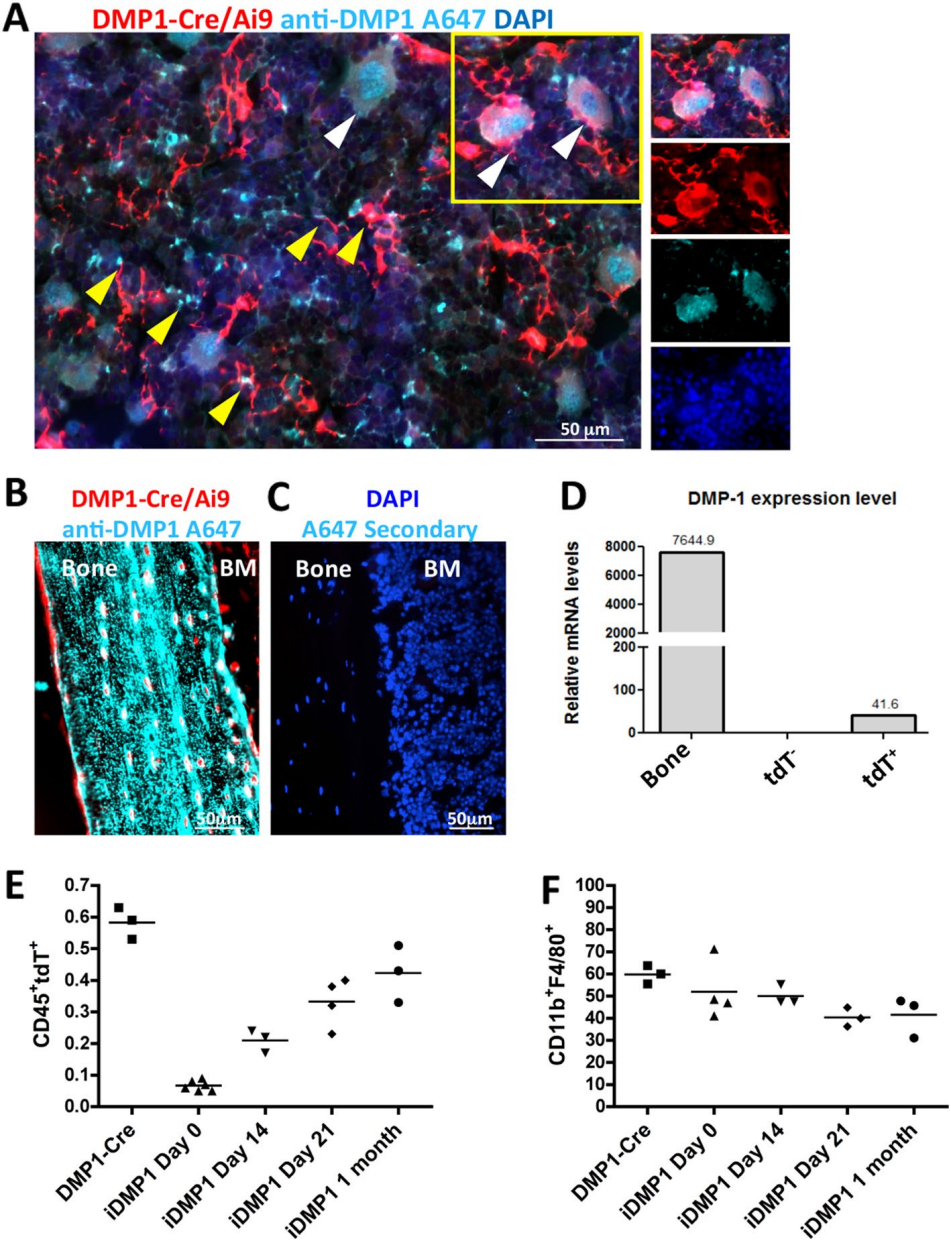
Endogenous DMP1 expression and lineage tracing of DMP1 in bone marrow. (**A**,**B**) Femoral frozen sections from DMP-Cre/Ai9 (red) stained with anti-DMP1 antibody (cyan) in bone marrow (**A**) and cortical bone as positive control (**B**). White arrows indicate colocalization of DMP1-Cre labeled macrophages with DMP1 protein. Yellow arrows indicate DMP1-Cre labeled stromal cells with colocalization of DMP1 protein or within close proximity to secreted DMP1 protein. (**C**) Secondary only staining in a Cre− littermate as negative control. Blue-DAPI (**D**) Relative mRNA expression of *DMP-1* from sorted tdT^−^ and tdT^+^ bone marrow cells. RNA from bone was used as positive control. Representative experiment shown, n = 2. (**E**) Quantification of CD45^+^tdT^+^ cells by flow cytometry in lineage traced 6 week old Tamoxifen injected iDMP1-Cre/Ai9 animals, n = 3–5. (**F**) Quantification of percent CD11b^+^F4/80^+^ that are CD45^+^tdT^+^ cells in lineage traced iDMP1-Cre/Ai9 animals, n = 3–4. SEM is shown in graphs.

To evaluate endogenous DMP1 levels in BM, immunostaining of DMP1 protein was done on DMP1-Cre/Ai9 femurs (Fig. 6A–C). Punctate DMP1 protein was seen in close contact with tdT^+^ cell processes including colocalization (yellow arrows Fig. 6A). Large tdT^+^ hematopoietic cells resembling macrophages expressed cytoplasmic and nuclear DMP1. These tdT^+^DMP1^+^ cells were frequently surrounded by the processes of tdT^+^ cells (white arrows Fig. 6A and inset). Cortical bone, which highly expresses DMP1, was used as positive control (Fig. 6B). Importantly, *DMP-1* transcripts were found in tdT^+^ sorted cells when compared to tdT^−^ sorted population (Fig. 6D).

Since DMP1-Cre/Ai9 is a historical marker of DMP1 and its descendants we wanted to determine if DMP1-expression in CD45^+^ cells were present in adult mice. We utilized inducible DMP1-CreERT2/Ai9 mice (iDMP1) and compared marker expression of CD45^+^tdT^+^ population in bone marrow 14 days after Tamoxifen (Tx) with DMP1-Cre/Ai9 mice (Supplemental Fig. 9A). Similar distribution of myeloid markers within CD45^+^tdT^+^ was seen between both DMP1-Cre/Ai9 and iDMP1. Lineage tracing of iDMP1 animals demonstrated presence of CD45^+^tdT^+^ cells 1 month after labeling (Fig. 6E and Supplemental Fig. 9B) with very little change in CD11b^+^F4/80^+^ expression (Fig. 6F). tdT^+^ cells were also evident at 1 month post Tx injection in thymus (Supplemental Fig. 9C,D).

### Bone marrow resident DMP1-Cre/Ai9 labeled cells express RANKL and interact with B cells

**Figure 7:**
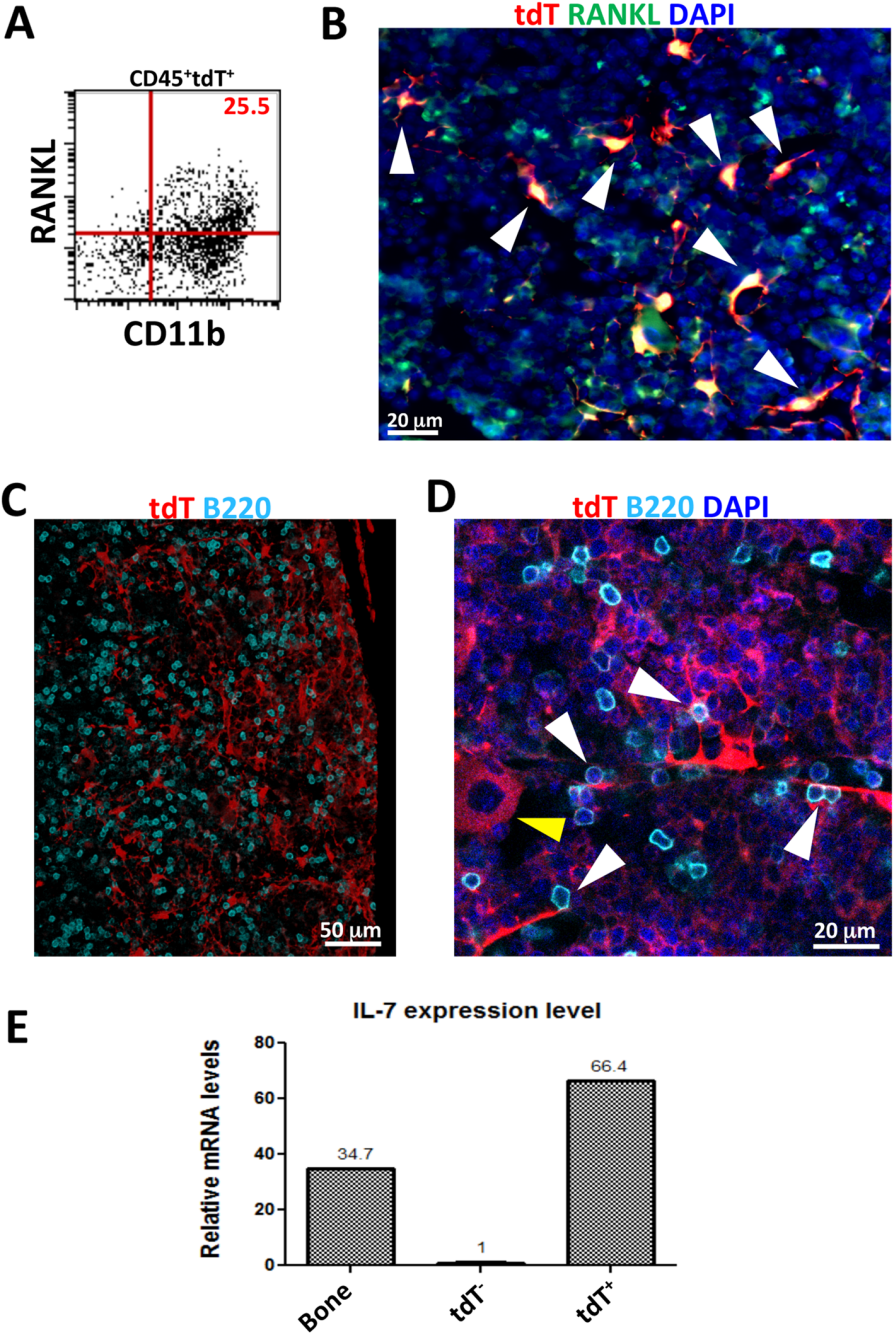
DMP1-Cre/Ai9 labeled cells express RANKL and contact B cells. (**A**) Flow cytometric analysis of RANKL expression in CD45^+^tdT^+^ parent population from bone marrow flush. (**B**) Bone marrow from femoral section stained with antibody to RANKL. Red–tdT, Green-RANKL, Blue-DAPI. White arrows—stromal cells double positive for RANKL and tdT. (**C**) Frozen histology section (20 μm) from femur of Dmp1-Cre/Ai9 animal stained for B220 and imaged by confocal microscopy. Z stack is shown. Red-tdT, Cyan-B220. (**D**) Confocal imaging of bone marrow demonstrates contacts between DMP1-Cre/Ai9 stromal cells and B cells (white arrows). Yellow arrow—tdT^+^ hematopoietic cell. Red-tdT, Cyan-B220, Blue-DAPI (**E**) Relative mRNA expression of *IL7* from sorted tdT^−^ and tdT^+^ bone marrow cells. RNA from bone was used as positive control, representative experiment shown, n = 2.

Matrix embedded osteocytes have been proposed as the main source of RANKL, which is critical for osteoclastogenesis, and indirectly supports B cells^63^. However, RANKL from adiponectin-Cre lineage stromal cells in BM has recently been identified to support osteoclastogenesis in some regions^64^. BM analyzed by flow cytometry for RANKL expression demonstrated 25.5% of CD45^+^tdT^+^ cells were RANKL^+^, all of which expressed CD11b (Fig. 7A). By histology, tdT^+^ BM cells showed co-expression of RANKL (Fig. 7B, white arrows). RANKL expression was also seen in the majority of tdT^+^ cells in thymus (Supplemental Fig. 10).

CXCL12^+^ cells have been shown to interact with B cells. We investigated if BM DMP1-Cre/Ai9 labeled cells have interactions with B cells^13^. Confocal microscopy identified B220^+^ cells in contact with tdT^+^ stromal cells (Fig. 7C,D, white arrows). We also detected large hematopoietic tdT^+^ cells (Fig. 7D, yellow arrows). tdT^+^ sorted cells analyzed for expression of the B cell supporting cytokine *IL-7* demonstrated levels higher than from bone (Fig. 7E).

## Discussion

We have shown that 10 kb DMP1-Cre/Ai9 labels rare subsets of hematopoietic and stromal cells in the BM. The hematopoietic tdT^+^ population is primarily myeloid, heterogenous based on marker expression, and has macrophage characteristics with a subset representing EIMs. These cells express DMP1 during adulthood and have limited expansion capacity. A rare hematopoietic DMP1-Cre tdT^+^ population also exists in adult spleen, peripheral blood and thymus. We have also demonstrated subsets of DMP-Cre tdT^+^ cells that express CXCL12 and RANKL. One limitation is that we have not confirmed that tdT^+^ CXCL12^+^ cells also co-express RANKL.

The CXCL12–CXCR4 signaling axis has been well documented for HSC, B cell and T cell survival, mobilization and/or differentiation (reviewed in^65^). This interaction is necessary for proper BM hematopoiesis and T cell development in thymus. Differences in cell surface markers from BM and thymus CD45^+^tdT^+^ cells most likely represent the different niches within these organs. Myeloid cells are more represented in BM and T cells in thymus. Expression of CXCR4 in both BM and thymus CD45^+^tdTomato^+^ cells suggest direct interaction with CXCL12^+^ stromal cells. Thymic macrophages account for around 0.1% of cells in thymus, are heterogenous and can be identified using various markers including CD11b and F4/80^42,66^. We report here a heterogenous population of tdT^+^ myeloid cells in thymus accounting for approximately 0.1% of thymic cells. Thymic atrophy seen in DMP1/DTR animals^39^ was most likely due to a direct effect from ablation of DMP1-lineage cells in the thymus and unrelated to osteocyte ablation.

G-CSF-mediated mobilization of HSCs is caused by down regulation of CXCL12 and VCAM1^67,68^. Depletion of BM CD169^+^ macrophages has also shown reduction of CXCL12 levels within the BM and egress of HSCs, but this depletion model targets not only macrophages within the HSC and mesenchymal niche^62^ but also EIM^52^. Since the CXCL12^+^ tdT^+^ stromal population forms a niche with tdT^+^ macrophages that express CD169 and VCAM1, it is possible that tdT^+^ macrophage depletion upon G-CSF treatment is due to this direct interaction between both cell types. In recent years, several groups have used Image Stream analysis of EIM macrophages and found that EIM are heterogenous^69–71^. EIM can have variable expression of key markers including CD11b, Ly6G and Ly6C. These subpopulations of EIM have been hypothesized to provide distinct functions during different stages of erythroblast maturation^72^. We have demonstrated a tdT^+^ myeloid population that is enriched when processing for EBIs and express EIM markers (Fig. 4). We have also found that both the Ly6G^−^ and Ly6G^+^ fractions show a decrease in VCAM and CD169 expression after G-CSF treatment (Fig. 5F–I) which is consistent with the hypothesis that EIM are heterogenous.

We observed that DMP1-Cre/Ai9 labeled cells in BM include a stromal population. Histologically most tdT^+^ cells had CXCL12 expression suggesting these represent a subset of CAR cells^32^. This is consistent with a previous study that demonstrated Cxcl12-GFP expression in 69% of DMP1-Cre/Ai9 labeled stromal cells^32^. We were able to expand these cells in culture, but only under non-standard conditions where bone tissue was present to support growth. Notably, in our previous study using iDMP1 mice we demonstrated the presence of labeled stromal cells that did not contribute to typical stromal colonies and showed limited expansion capacity in vitro^33^. Single cell RNAseq data also detects *Dmp1* in a small subset of adipogenic CAR cells, albeit at lower levels than osteoblasts^73,74^. Recently it has been shown that adipogenic CAR cells have direct interactions with hematopoietic fractions within BM. Using Image Stream analysis, Matsushita et al. showed adherence of CD45^+^ cells and fragments to Cxcl12-GFP cells and cells double positive for CD45 and Cxcl12^14^. Furthermore, a fraction of Cxcl12^+^ hematopoietic cells expressed monocytic markers CD11b, F4/80, and Ly6G similar to what we show here for DMP1-Cre/Ai9 labeled cells (Fig. 1). Yu et al. has recently demonstrated that adipogenic CAR cells targeted by Adipoq-Cre express RANKL and interact with cells of the monocyte-macrophage lineage^15^. Furthermore, these cells have been shown to be the major contributor to M-CSF in the BM where they support resident macrophages and osteoclasts^16^. DMP1-Cre labeled cells resemble a subpopulation of Adipoq-Cre labeled adipogenic CAR cells due to their expression of RANKL and close proximity and support to resident macrophages.

Osteocytes and osteoblasts have been identified as the key source of RANKL for osteoclast differentiation. However, a unique interaction between B cell lymphopoiesis by IL-7 and by osteoblastic RANKL has been demonstrated in the literature (reviewed in^60^). In vitro, overexpression of RANKL in pre-B cells in combination with IL-7 was necessary to support B cell maturation^75^. It is possible that DMP1-Cre/Ai9 labeled cells contribute to a unique B cell-osteoclast progenitor niche that was previously thought to occur more from osteoblastic support. We have demonstrated DMP1-Cre/Ai9 tdT^+^ cells express RANKL, and express IL-7 (Fig. 7).

Our data indicates that the CD45^+^tdT^+^ cells are primarily myeloid and include tissue-resident macrophages that can be depleted via targeting them directly with clodronate liposomes, or indirectly via chemotherapeutic myeloablation, or niche disruption. However, several recent studies have highlighted problems with using flow cytometry techniques to characterize macrophages that were not fully recognized when we undertook these studies that could lead to misassignment of tdT^+^ cell identity. Pettit’s group has shown that intact macrophages are not present in ex vivo cell hematopoietic preparations^40^. Imaging flow cytometry revealed that remnants of macrophages are bound to other hematopoietic and non-hematopoietic cell types^40^, consistent with a previous study demonstrating this phenomenon in lymph nodes^76^. Because of this artifact, it has been concluded that F4/80^+^Ly6G^+^ cells initially thought to represent EIMs in BM preparations are F4/80^+^ macrophage remnants bound to other cell types including Ly6G^+^ neutrophils. Considering the report by Pettit’s group, this may also partially explain the heterogeneity in marker expression in our tdT^+^ population. Nonetheless, our Image Stream data demonstrates the presence of cells with full coverage of cytoplasmic tdT distribution indicating that not all labeled cells are macrophages prone to fragmentation. Another challenge that arises when studying macrophages is their ability to phagocytose cells^76,77^. Because of the close association between tdT^+^ stromal cells and macrophages, phagocytosis or efferocytosis of stromal cell fragments by resident macrophages could be occurring leading to tdT^+^ reporter expression in myeloid cells. Likewise, our Image Stream data and histology of tdT^+^ cells with cytoplasmic tdT expression, and large non stromal tdT^+^ cells with reporter distribution throughout the cell and endogenous DMP1 expression are evident histologically (Fig. 6A), suggesting this is not the main mechanism by which tdT^+^ myeloid cells are generated. Furthermore, methods to isolate EBIs with limited shear stress or disaggregation techniques cause an increase in these cell numbers (Fig. 4). In order to fully understand the heterogeneity of DMP1-Cre labeled cells in BM, higher resolution techniques such as single cell RNAseq would be useful, however given that macrophages are lost during processing, and it is possible that the stromal population with extensive processes do not survive isolation efficiently either, an in situ method would be preferable.

Previously we have reported DMP1 expression in bone using four transgenic animals^34^. The 8 kb DMP1 promoter driven GFP mouse efficiently labels only osteocytes by histology and flow cytometry confirmed the lack of DMP1-GFP expression in BM flushes (^34^ and data not shown). Specificity of DMP1-GFP to osteocytes is likely due to lower sensitivity of GFP versus Cre/reporter system. Both the 8 kb DMP1-Cre and 10 kb DMP1-Cre lines when crossed with Ai9 label osteocytes, osteoblasts, muscle and BM cells. We have not compared 8 kb DMP1-Cre directly with 10 kb DMP1-Cre in this current study. The inducible model, 10 kb DMP1-CreERT2, also efficiently targets osteoblasts, osteocytes, some muscle cells and cells in the BM when combined with a sensitive reporter like Ai9, but is more restricted with Rosa26-based LacZ staining^34^. A bicistronic DMP1-Cre knock-in mouse has recently been created^78^. This mouse when crossed with Ai14 showed DMP1-Cre reporter expression in bone as well as soft tissues including skeletal muscle and intestinal cells. In the future it would be worthwhile to determine if DMP1-Cre activity is also present in the bone marrow in this knock-in line, but given that our immunostaining data and the use of the inducible model indicate endogenous DMP1 expression in marrow resident cells we would predict it would be present.

In conclusion we have analyzed 10 kb DMP1-Cre/Ai9 labeled bone marrow and extramedullary hematopoietic organs for tdT^+^ cells and found a heterogenous population of hematopoietic and stromal cells outside of the mature osteoblast/osteocytes. tdT^+^ cells in BM and thymus express RANKL where they may provide a supporting role for osteoclasts and T cells. tdT^+^ cells in the BM also express *IL-7* that can affect differentiation of B cells^13^. Therefore, the microenvironmental support from DMP1-Cre/Ai9 labeled cells should be considered when using DMP1/Cre animals in the future when studying bone biology.

## Methods

### Mice

All experiments and procedures were approved by the Institutional Animal Care And Use Committee (protocol number 200271-1023) at the University of Connecticut Health. All methods were performed in accordance with the relevant guidelines and regulations and in accordance with ARRIVE guidelines. All animals were housed in a temperature-controlled environment on a 12 h light dark cycle and fed rodent chow and water ad libitum. The following transgenic mouse lines were previously described: 10kbDmp1Cre (DMP1-Cre)^19,34^ and Dmp1CreERT2 (iDMP1)^79^. Ai9 tdTomato (tdT) reporter mice were purchased from Jackson Laboratories (stock#007909)^80^. Cre recombination was induced with (75 mg/g) tamoxifen (Sigma-Aldrich Corp., St. Louis, MO, http://www.sigmaaldrich.com) by intraperitoneal injection. Time zero was considered 2 days following tamoxifen injection. Experiments were done with animals 8–16 weeks of age unless otherwise stated. Timed pregnancies were done for experiments requiring embryos. Male and female age matched littermate Cre negative controls were used in all experiments with a n of 3–5 per experimental group. For experiments with treatment groups, animals were divided randomly and analyzed blindly with number allocations.

### Irradiation

Adult mice received total body gamma irradiation with lethal dose of 950 cGy using a ^137^Cs source (gammaCell/Best Theratronics).

### In vivo macrophage depletion

PBS or Clodronate loaded liposomes (Liposoma B.V., Amsterdam, The Netherlands, ClodronateLiposomes.org) were injected retro-orbital at 10 ml/g mouse and animals sacrificed 36 h later^10^. Fluorouracil (5-FU) (Sigma-Aldrich Corp., St. Louis, MO) was injected intraperitoneal at a dose of 150 mg/kg and animals sacrificed 4 days later^81^.

### G-CSF mobilization

Human G-CSF (Isokine-ORF Genetics) was injected subcutaneous twice a day for 3 days at a dose of 50 mg/kg^82^. Efficiency of mobilization was monitored by analyzing peripheral blood and spleen single cell suspensions for hematopoietic stem cells by flow cytometry for Lineage^−^Sca1^+^C-kit^+^ (LSK).

### LPS

Three hours before sacrifice, 25 mg LPS from E. coli Serotype 055:B5 (Alexis biochemical, EnzoLife) in a 100 ml volume was injected intraperitoneal.

### Histology

Soft tissues were fixed for 12–24 h and bones were fixed for 3 days in 4% paraformaldehyde (PFA). Bones were then decalcified in 14% EDTA for 2 weeks. Tissues were placed overnight in 30% sucrose prior to embedding in cryomatrix (Shandon). Frozen sections (7–20 mm) were cut using a tape transfer system and a Leica cryostat and then glued to glass slides with Norland optical adhesive 61 (Norland Products). All sections were hydrated in PBS prior to mounting and stained with 4′,6-diamidino-2-phenylindole (DAPI) prior to coverslipping in 50% glycerol.

### Immunohistochemistry

Primary and secondary antibody information can be found in Supplemental Table 1. For DMP1 and F4/80 staining, hydrated frozen sections were incubated with 0.3% triton and then blocked in 10% donkey serum. Primary antibody staining was overnight at 4 °C followed by appropriate secondary staining for one hour at room temperature. For directly conjugated antibodies (CXCL12, RANKL, B220 and Ter119), hydrated sections were incubated in 0.3% triton and then with conjugated antibodies overnight at 4 °C in 2% FBS. For RANKL staining, Image-iT FX signal enhancer (Invitrogen, 136933) was used according to manufacturer’s protocol. Images were acquired on a Axioscan Z.1 (Zeiss) or Zeiss Axio Observer Z1 LSM 880 (Zeiss).

### Flow cytometry

Soft tissues including spleen, liver and thymus were made into single cell suspensions by rubbing organs between two frosted glass slides and then filtering through a 100 mm cell strainer. Bone marrow was prepared by flushing femurs and tibias with a 25G needle and Staining Media (SM) containing 2% Newborn Calf Serum, 1 × HBSS, and 1 mM HEPES. Cell pellets were lysed with Red Blood Cell Lysing Buffer (Sigma) for 5 min and then washed and filtered through 70 mm Nitex. Peripheral blood was collected into 0.5 M EDTA and RBCs sedimented in Dextran-500 (Sigma, 31392) as previously described^83^. 5 million cells per 100 ml antibody cocktail were stained on ice for 45 min. Antibodies used for flow cytometry are listed in Supplemental Table 2. Cells were washed with SM and resuspended in Sytox Blue Dead Cell Stain or stained with UV live/dead (both from Molecular Probes). Multiparameter analysis was done on a BD-LSR II and cell sorting on a BD-FACS ARIA II (BD Biosciences; San Jose, CA, USA) with FACS DIVA software. tdT negative gate was set using littermate controls negative for Cre. Cell sorting was done using the 100 mm nozzle and sorted into tubes containing 20% FBS (Atlanta Biologics). All sorts were reanalyzed for purities of greater than 95%. FACS data was analyzed using BD CellQuest Pro™ Software or FlowJo version 10 and then exported into Canvas™X (ACD Systems; Miami, FL, USA) for figure rendering. For flow cytometry with imaging, cells were fixed in 2% PFA after staining and were analyzed on a Amnis Imagestream^X^Mark II (Luminex).

### Thymic digestion for flow cytometry

Thymic lobes were separated and digested as previously described^43^. Briefly, thymic capsules were knicked with scissors and placed in RPMI-1640 (Gibco) for 30 min with agitation to remove thymocytes. Thymi were incubated with 0.125% Collagenase D (Roche) and 0.1% DNAse I (Roche) for 10 min for a total of 3 times with supernatant collected and pooled for each digest. Pooled digests were washed and filtered through 70 mm Nitex.

### Aire staining

Single cell suspensions of bone marrow and thymi were stained for CD45 and Fixable Viability Dye eFluor780 (ebioscience). Cells were fixed and stained according to manufacture protocol using Flow Fix and Perm Buffer Kit (R&D Systems, FC009) and anti-Aire (Thermofisher, 14-5934-82) with a rabbit anti-rat FITC secondary. For histology, hydrated thymic sections were permeabilized with 0.5% triton for 2 h, incubated for 10 min in Power Block (BioGenex, HK085-5K) and then incubated overnight at 4 °C with anti-Aire in 0.3% triton followed by rabbit anti-rat A488 secondary (Jackson ImmunoResearch, 312-545-003).

### Phagocytosis assay

Five million BM cells were resuspended in 1 ml RPMI-1640 (Gibco) supplemented with 10% FBS, 1 × HEPES and 0.5 ml Fluoresbrite YG 1 mm beads (Polysciences, Inc) and then incubated at 37 °C for 1 h. Cells were washed three times with PBS and stained with cell surface markers as described.

### Isolation and flow cytometry of erythroblast island macrophages (EIM) and intact islands

Initial evaluation of tdT^+^ EIM was done by serum overlays and percoll (Sigma) gradients as previously described^58,59^. Methods to obtain intact blood islands with EIM were modified from^55,56^. One femur was flushed and processed as described above to be used as a control and for cell counts. The second femur was flushed using an 18G syringe with 3 ml IMDM, 20% FCS and 3.5% sodium citrate which corresponds to approximately 10 million cells/ml. Aggregates were gently titrated twenty times with a Pasteur pipet and then 200 ml (approximately 2 million cells) was stained with flow antibodies for 2 h at room temperature and then resuspended in 3.5 × volume of SM containing DAPI or Sytox blue viability dye. Gating for singlets and multiplets was done on live cells gated for SSC-area vs SSC-height with blood islands being multiplets double positive for Ter119 and F4/80^55^. Antibodies chosen for EIM identity included F4/80, VCAM1, CD169, ERHR3 and Ly6G^52^. For experiments with low BM cellularity, titrated BM was spun for 3 min at 300*g* and resuspended in the appropriate volume for EIM staining.

### Cytospins

Sorted cells were washed in PBS and resuspended in 30% FBS. A volume of 0.3 ml was placed in cytospin chambers (Shandon, ThermoScientific; Pittsburgh, PA, USA) with pretreated glass slides and centrifuged at 450 rpm in a Shandon cytospin. Slides were stained with Wright-Giemsa kit HEMA 3 (Fisher Scientific; Hampton, NH, USA) according to the manufacturer’s protocol. Slides were photographed using a 20 × objective Zeiss Axiocam (Carl Zeiss; Thornwood, NY, USA).

### Cell culture

BM aspirates were plated at a density of 2.5 million cells/well in 24 well plates and were grown in MEMalpha (Invitrogen), 20% FBS (Atlanta Biologics) with one 2 week old B6 femur cut longitudinal to open the marrow cavity. Half media changes were done every second day. Cells were detached from the dish using 0.05% Trypsin–EDTA for flow cytometry.

### Gene expression

RNA was extracted from sorted cells using Trizol solution and converted to cDNA with Improm-II Reverse Transcription System (Promega). TaqMan Gene Expression Assays used for real-time PCR included Dmp1 (Mm00803833_g1), Il7 (Mm00434291_m1), Gapdh (Mm999999915_g1).

### Statistical analyses

Unpaired *t* test (GraphPad Prism) was used to evaluate the significance of differences between two groups. All data are presented as mean ± SEM. All experiments included at least n = 3, while RNA data presented in Fig 6D and 7E had n=2 as indicated within the figures.

### Ethics approval and informed consent

No human subjects were recruited for these studies. All animal experiments and procedures were approved by the Institutional Animal Care And Use Committee (protocol number 200271-1023) at the University of Connecticut Health and is reported according to ARRIVE guidelines.

### Supplementary Information

Supplementary Information.

## Data Availability

Data that support the findings of this study are available upon request to the corresponding authors.
