# Supplementary material for: Hematopoietic and stromal DMP1-Cre labeled cells form a unique niche in the bone marrow
Source: Sci Rep. 2023 Dec 16;13:22403. doi: 10.1038/s41598-023-49713-x (PMC10725438; doi:10.1038/s41598-023-49713-x)
Supplement: Supplementary file 1 — Supplementary Information. [file 41598_2023_49713_MOESM1_ESM.docx]

**
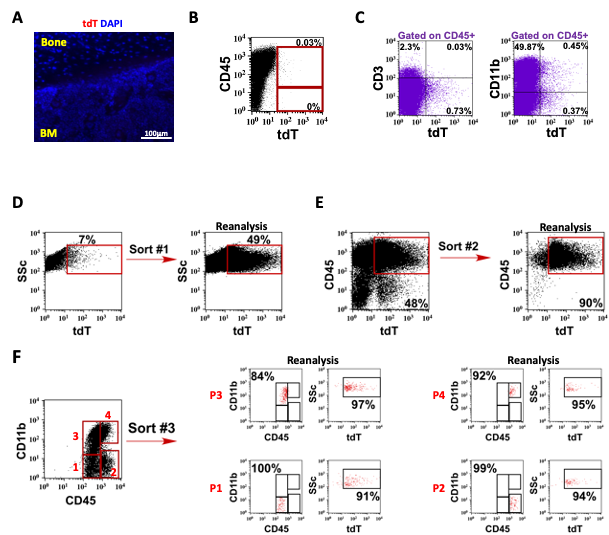
**

**Supplemental Figure 1**

**Dmp1-Cre^-^/Ai9 controls and triple FACS sorting to obtain highly purified CD45^+^tdT^+^CD11b^-/+^ subsets used in cytospins**

**(A)** Frozen femoral bone section of Dmp1-Cre^-^/Ai9 littermates imaged for tdT and DAPI **(B)** Flow cytometric analysis of Dmp1-Cre^-^/Ai9 littermates for CD45 and tdT expression. **(C)** Flow cytometric analysis of Dmp1-Cre^+^/Ai9 bone marrow gated on CD45+ cells for CD3 and tdT and CD11b and tdT. **(D)** Sort 1 and reanalysis of tdT^+^ versus side scatter (SSC). **(E)** Sort 2 and reanalysis of CD45^+^tdT^+^cells. **(F)** Sort 3 and reanalysis of four populations of CD11b CD45 expressing tdT^+^.


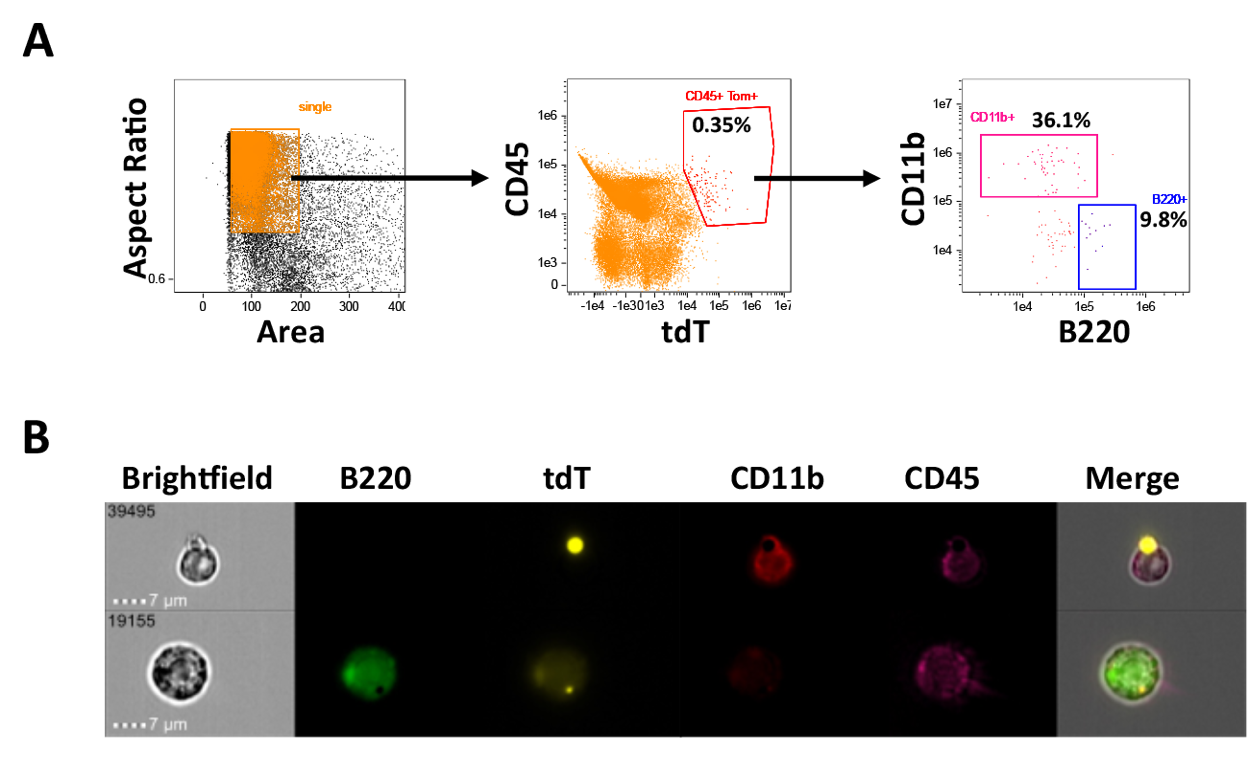


**Supplemental Figure 2**

**Image Stream Analysis used in Figure 1D.**

**(A)** Gating strategy used to identify CD45^+^tdT^+^ cells in Image Stream. **(B)** Rare examples of tdT^+^ fragments in CD11b and B220 cells by Image Stream.


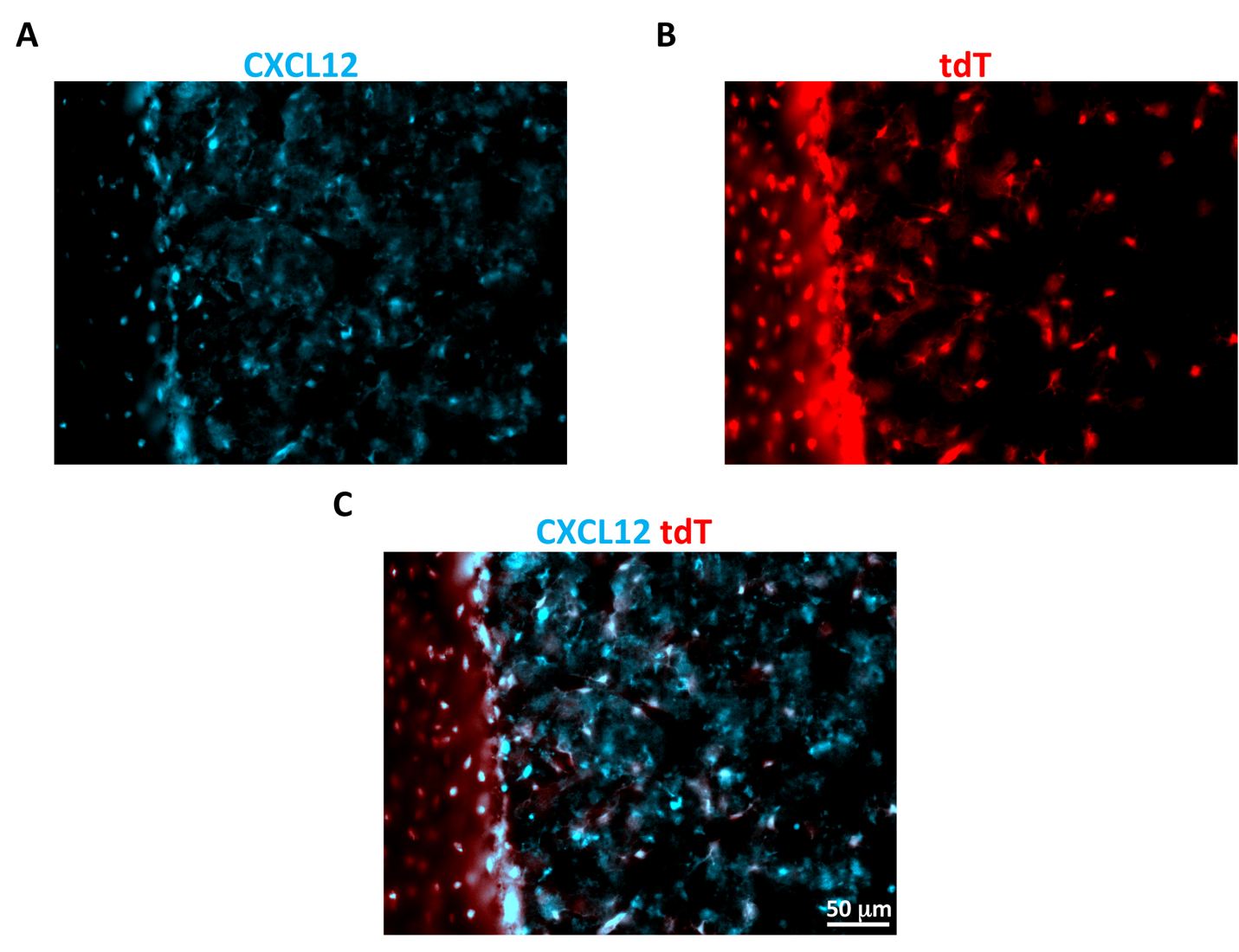


**Supplemental Figure 3**

**Individual channels of CXCL12 staining of Dmp1-Cre/Ai9 labeled bone marrow**

**(A-C)** Individual slice from z stack for CXCL12 staining and tdT reporter. **(A)** CXCL12 staining (cyan) and **(B)** tdT (red) from Dmp1-Cre/Ai9 femoral frozen section. **(C)** Merged image.


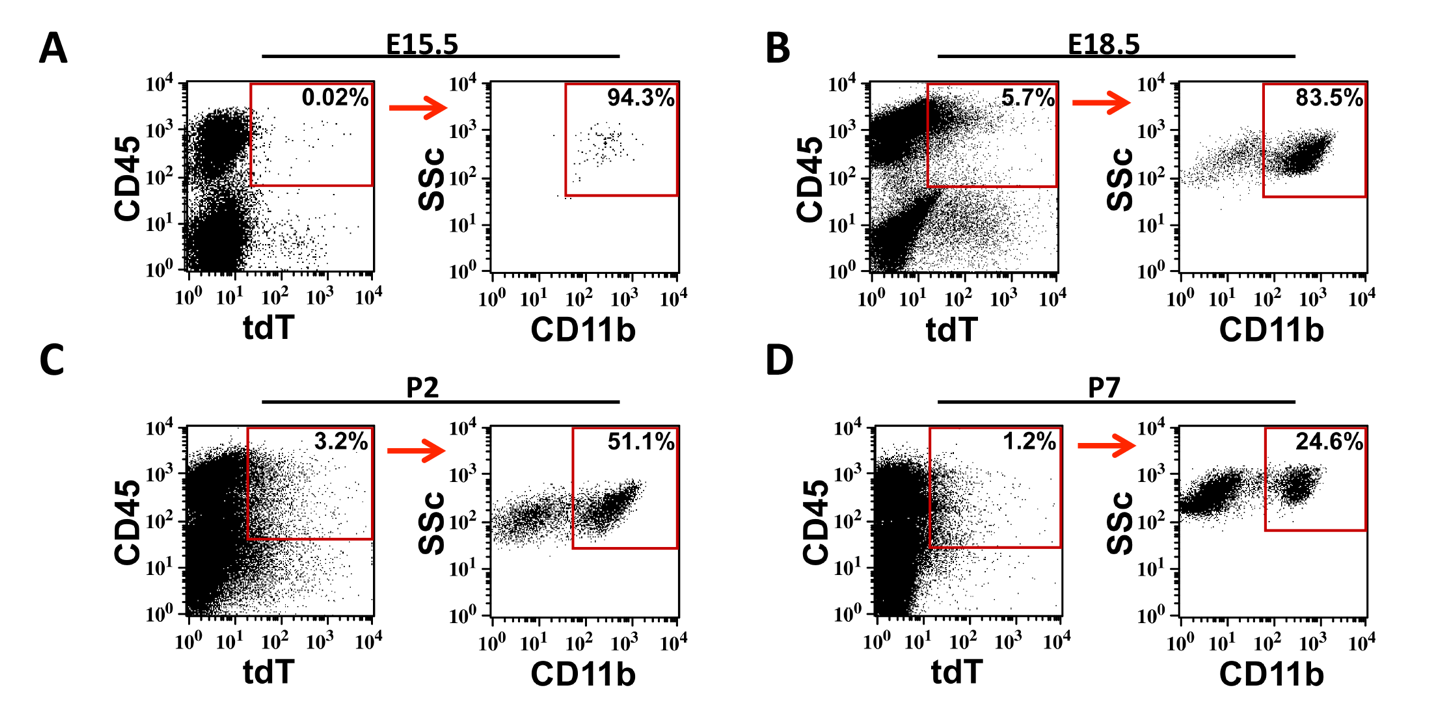


**Supplemental Figure 4**

**Hematopoietic Dmp1-Cre/Ai9 labeled cells in bone marrow during development**

**(A-D)** Pooled bone marrow flushes from embryonic (E)15.5 **(A)**, E18.5 **(B)**, post-natal (P) day 2 **(C)** and P7 **(D)** were analyzed by flow cytometry for CD45 and tdT expression. Cells gated on CD45^+^tdT^+^ were analyzed for CD11b expression. None detected – N/D. n=2-4.


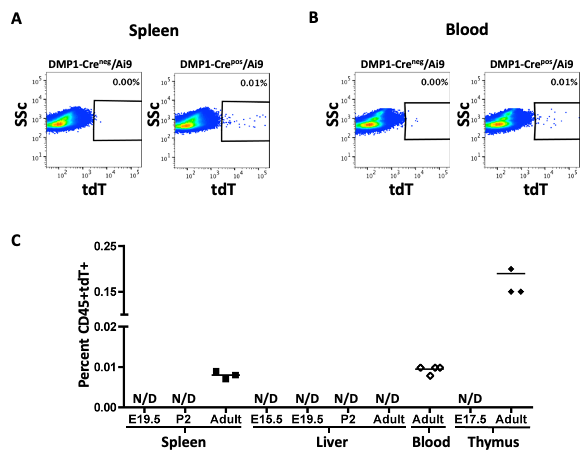


**Supplemental Figure 5**

**Analysis of Dmp1-Cre/Ai9 labeled cells in secondary hematopoietic organs**

**(A)** Flow analysis of spleen and peripheral blood from 5 week old Dmp1-Cre/Ai9 animals **(B)** Quantification of CD45^+^tdT^+^ cells by flow cytometry from spleen, liver, blood and thymus during development and in adult Dmp1-Cre/Ai9 animals. N/D = Not detected. n=2-5.

**
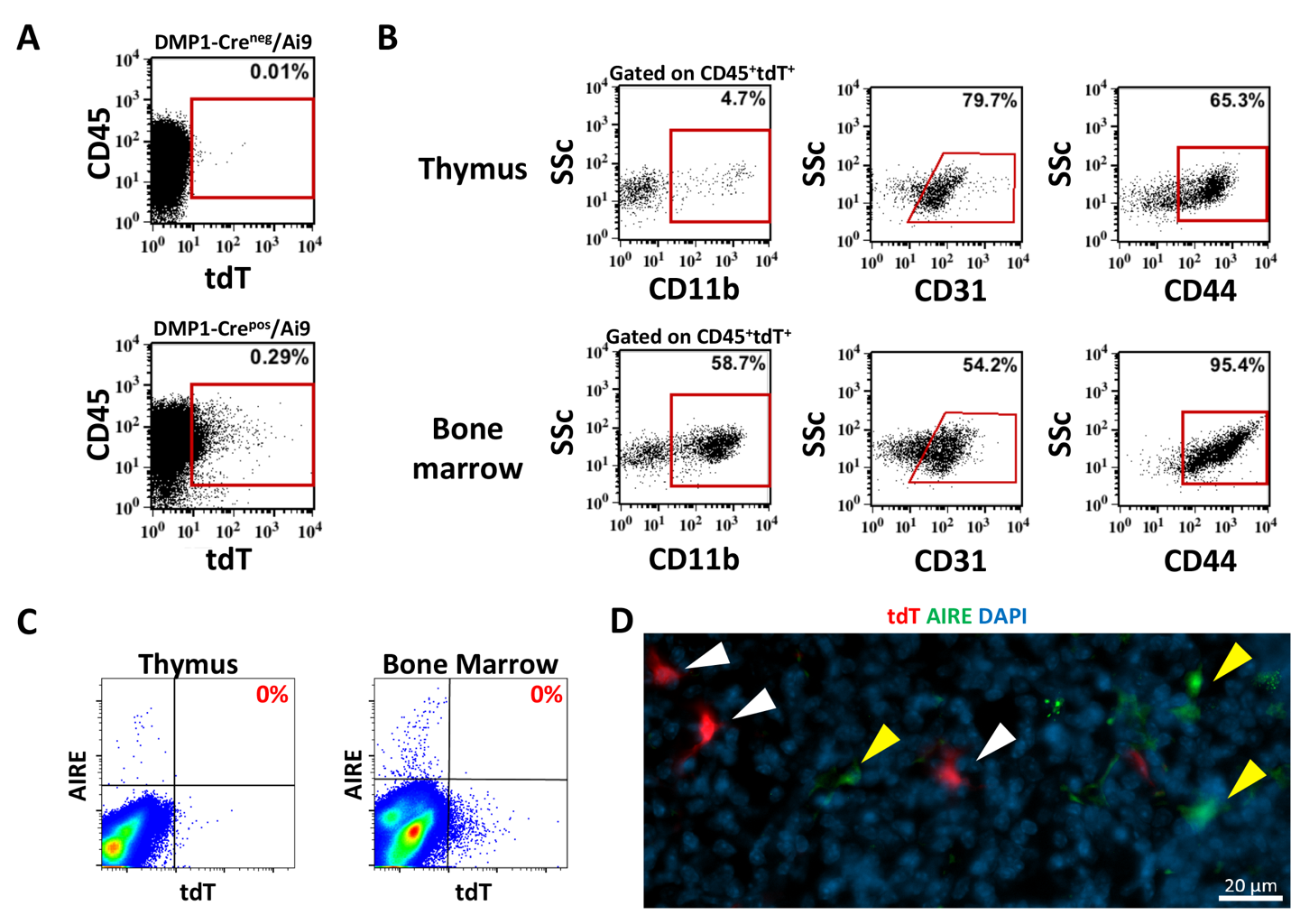
**

**Supplemental Figure 6**

**Dmp1-Cre/Ai9 labeled cells in thymus are not medullary thymic endothelial cells (mTECs)**

**(A)** Thymi from 8 week old Cre- and Cre+ animals were enzymatically processed to obtain mTECs. Digested fractions were analyzed for CD45 and tdT expression. **(B)** CD45^+^tdT^+^ cells were analyzed for CD11b, CD31, and CD44 expression and compared to bone marrow, n=3. **(C)** Flow cytometry plots of thymus and bone marrow for AIRE and tdT expression from 4 month old Dmp1-Cre/Ai9 animals. **(D)** Histological frozen section of Dmp1-Cre/Ai9 thymus immunostained for AIRE. White arrows- tdT^+^ cells (red), yellow arrows- AIRE^+^ cells (green), DAPI (blue).


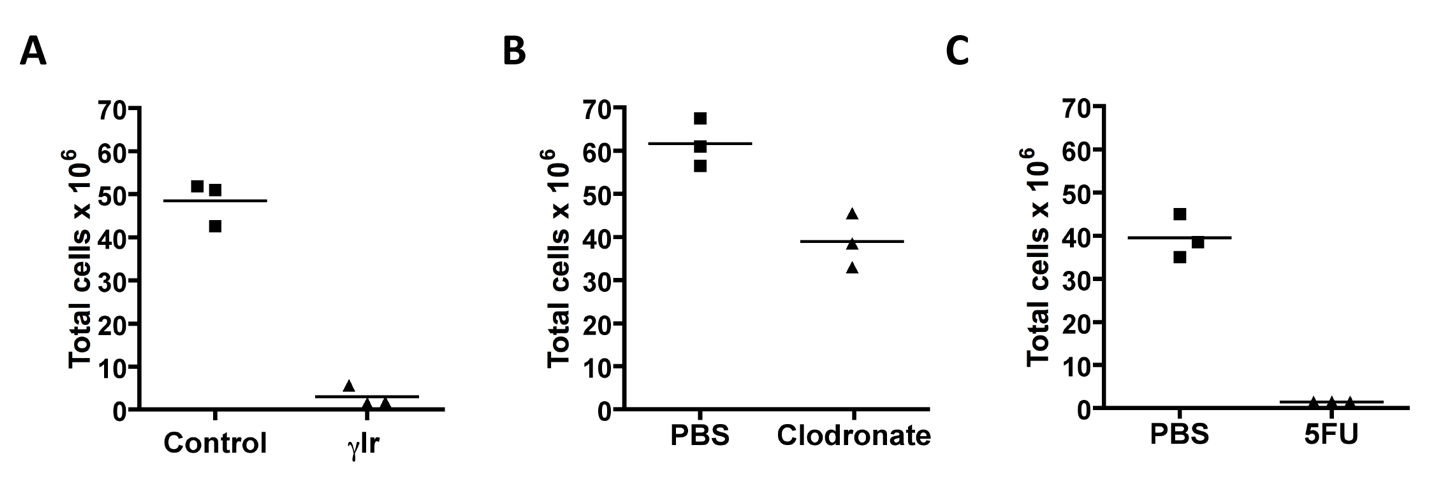


**Supplemental Figure 7**

**Bone marrow cellularity in challenged Dmp1-Cre/Ai9 animals**

**(A-C)** Bone marrow cellularity in controls and gamma irradiated **(A**), clodronate **(B),** and 5FU **(C)** treated animals, n=3.


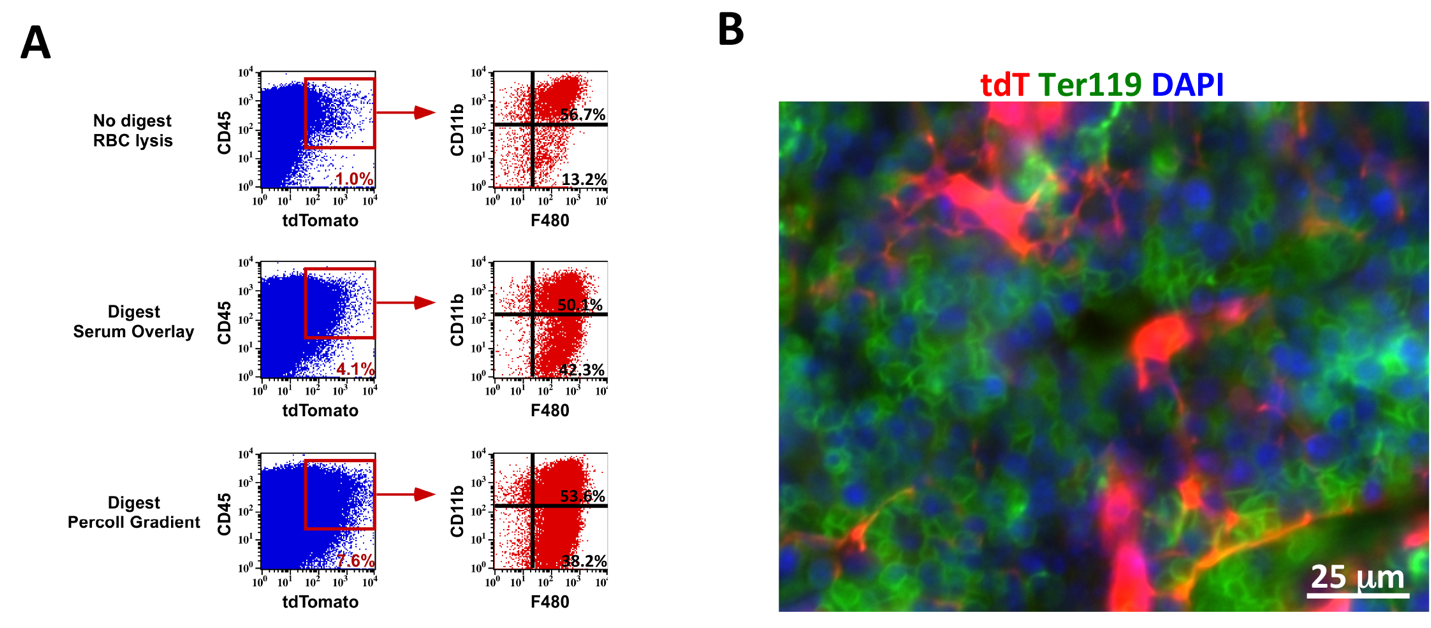


**Supplemental Figure 8**

**Dmp1-Cre/Ai9 labeled hematopoietic fraction is enriched in erythroid blood islands and express key markers of erythroid island macrophages (EIM)**

**(A)** Digested bone marrow from 12 week old Dmp1-Cre/Ai9 animals processed for EIM using serum overlays or percoll gradient was compared to traditional method of no digestion and RBC lysis. Enrichment of CD45^+^tdT^+^ cells occur in EIM preparations. CD45^+^tdT^+^ cells were gated and analyzed for CD11b and F4/80. **(B)** Histological frozen section of Dmp1-Cre/Ai9 bone marrow stained for Ter119 (green).


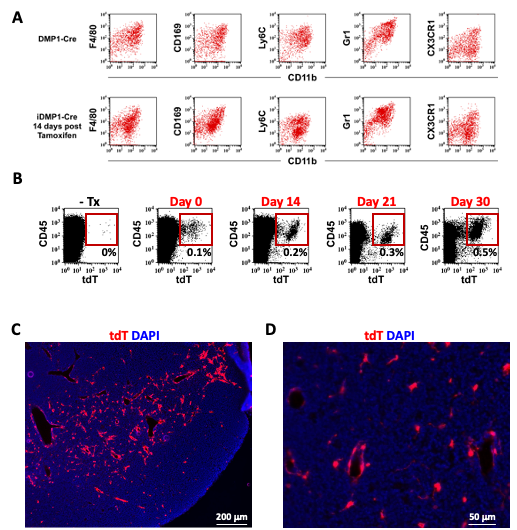


**Supplemental Figure 9**

**Inducible DMP1-Cre (iDMP1) labeling in bone marrow and thymus**

**(A)** Flow cytometric analysis of CD45^+^tdT^+^ bone marrow cells for myeloid markers in iDMP1-Cre/Ai9 animals 14 days after tamoxifen. Similar distribution of markers is seen when compared to DMP1-Cre/Ai9, n=2. **(B)** Lineage tracing of 6 week old iDMP1-Cre/Ai9 animals after Tx administration. Representative flow cytometric analysis of CD45^+^tdT^+^ bone marrow cells is shown, n=3-5. Mice treated without Tx is shown as a control. **(C)** Low (4x) and (**D)** high (20x) magnifications of thymus from iDMP1 animal 1 month after tamoxifen injection.


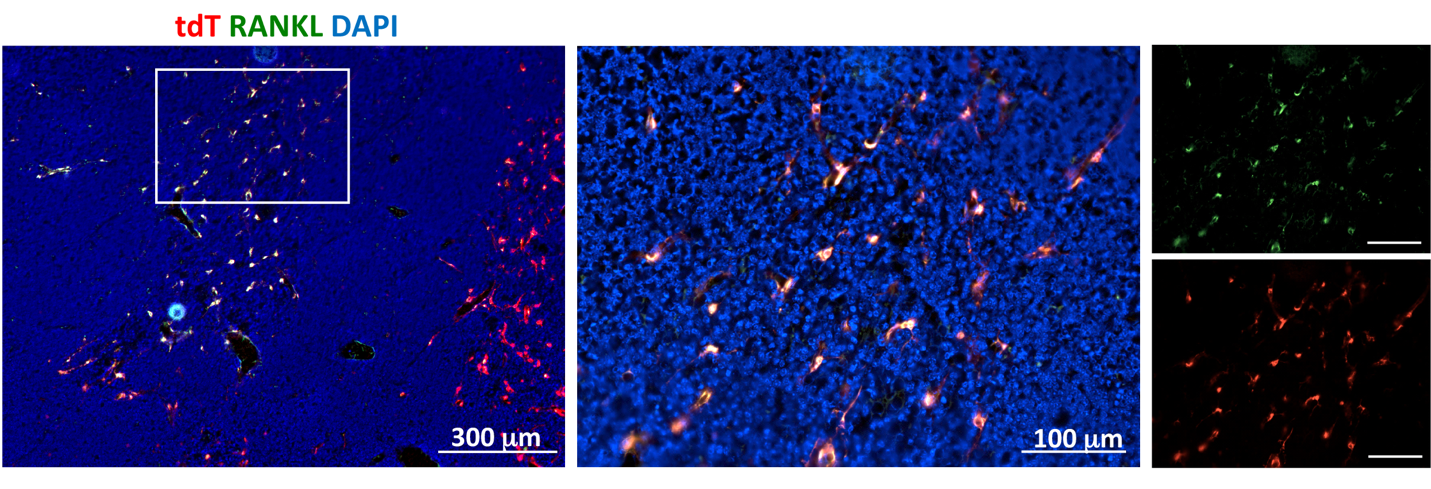


**Supplemental Figure 10**

**DMP1-Cre/Ai9 labeled cells in thymus express RANKL**

Frozen histology of DMP1-Cre/Ai9 thymus stained for RANKL. Merged and individual channels shown. Red–tdT, Green-RANKL, Blue-DAPI.

**Supplemental Table 1:** Antibodies for immunostaining

| **Primary**  **Antibody** | **Company** | **Secondary Antibody** | **Company** |
| --- | --- | --- | --- |
| DMP1  (1:50) | R&D Systems (AF4386) | donkey anti-sheep A647  (1:400) | Jackson ImmunoResearch (713-606-147) |
| F4/80  (1:100) | Cell Signaling Technology (712996) | donkey anti-rat A647  (1:400) | Jackson ImmunoResearch (712-607-003) |
| CD31  (1:200) | R&D Systems (AF3628) | donkey anti-goat A647  (1:400) | Jackson ImmunoResearch (705-605-147) |
| CXCL12/SDF1-APC  (1:50) | R&D Systems (IC350A) | N/A | N/A |
| TRANCE/TNFSF11/RANKL-Alexa Fluor 488  (1:50) | Novus Biologicals (NBP2-27389) | N/A | N/A |
| B220 APC (clone RA3-6)  (1:100) | eBioscience  17-0452-82 | N/A | N/A |
| Ter119 FITC (clone Ter119)  (1:100) | eBioscience  11-5921-82 | N/A | N/A |

**Supplemental Table 2:** Antibodies for flow cytometry

| **Antibody** | **Clone** | **Conjugation** | **Company** |
| --- | --- | --- | --- |
| CD45 | 30-F11 | APC, APCe780 | ebioscience |
| CD45 | 30-F11 | A700, BV711 | Biolegend |
| CD11b | M1/70 | FITC, APC, APCe780, e450 | ebioscience |
| CD11b | M1/70 | BV711 | Biolegend |
| F4/80 | BM8 | FITC, A700 | ebioscience |
| F4/80 | BM8 | BV510 | Biolegend |
| CD62L | MEL-14 | APCe780 | ebioscience |
| CCR2 (CD192) | 475301 | FITC | R&D Systems |
| Ly6C | AL-21 | A700 | BD Pharmingen |
| Ly6C | HK1.4 | BV711 | Biolegend |
| Ly6G | 1A8 | FITC, A700 | Biolegend |
| Ly6G (Gr1) | Gr1 | FITC | ebioscience |
| CX3CR1 | SA011F11 | BV605 | Biolegend |
| CXCR4 (CD184) | 2B11 | biotin | ebioscience |
| CD31 | 390 | biotin, e450 | ebioscience |
| CD44 | IM7 | biotin | BD Pharmingen |
| Ter119 | Ter119 | FITC, APCe780 | ebioscience |
| ER-HR3 | ER-HR3 | A488 | Novus |
| CD169 | SER-4 | e660 | ebioscience |
| VCAM1 (CD106) | 429 | e450 | ebioscience |
| CD51 | RMV-7 | biotin | Biolegend |
| RANKL (CD254) | 12A380 | A488 | Novus |
| B220 (CD45R) | RA3-6B2 | A700, APCe780 | ebioscience |
| Streptavidin | N/A | BV650 | BD Biosciences |
| Streptavidin | N/A | APCe780 | ebioscience |
